# Rectal Radiation Dose and Clinical Outcomes in Prostate Cancer Patients Treated With Stereotactic Body Radiation Therapy With and Without Hydrogel

**DOI:** 10.3389/fonc.2022.853246

**Published:** 2022-03-08

**Authors:** Palak Kundu, Eric Y. Lin, Stephanie M. Yoon, Neil R. Parikh, Dan Ruan, Amar U. Kishan, Alan Lee, Michael L. Steinberg, Albert J. Chang

**Affiliations:** ^1^ Department of Radiation Oncology, David Geffen School of Medicine at UCLA, Los Angeles, CA, United States; ^2^ David Geffen School of Medicine at UCLA, Los Angeles, CA, United States

**Keywords:** hydrogel, prostate cancer, SBRT (stereotactic body radiation therapy), radiation oncology, outcomes

## Abstract

**Background:**

Patients with prostate cancer treated with stereotactic body radiation therapy (SBRT) may experience gastrointestinal (GI) toxicity. The hydrogel may mitigate this toxicity by reducing the rectal radiation dose. The purpose of this study is to compare rectal radiation dose and GI toxicity in patients receiving prostate SBRT with and without hydrogel.

**Methods:**

Consecutive patients treated with SBRT between February 2017 and January 2020 with and without hydrogel were retrospectively identified. Baseline characteristics including prostate volume, rectal diameter, body mass index (BMI), age, pretreatment prostate-specific antigen (PSA), Gleason score, T-stage, and androgen deprivation therapy (ADT) usage were compared. Dosimetric outcomes (V40Gy, V36Gy, V32Gy, V38Gy, and V20Gy), rates of acute (≤90 days) and late (>90 days) GI toxicity, and PSA outcomes were evaluated for patients with and without hydrogel.

**Results:**

A total of 92 patients were identified (51 hydrogel and 41 non-hydrogel). There were no significant differences in baseline characteristics. Rectal V38(cc) was significantly less in the hydrogel group (mean 0.44 vs. mean 1.41 cc, p = 0.0002), and the proportion of patients with V38(cc) < 2 cc was greater in the hydrogel group (92% vs. 72%, p = 0.01). Rectal dose was significantly lower for all institutional dose constraints in the hydrogel group (p < 0.001). The hydrogel group experienced significantly less acute overall GI toxicity (16% hydrogel vs. 28% non-hydrogel, p = 0.006), while the difference in late GI toxicity trended lower with hydrogel but was not statistically significant (4% hydrogel vs. 10% non-hydrogel, p = 0.219). At a median follow-up of 14.8 months, there were no biochemical recurrences in either group.

**Conclusion:**

Hydrogel reduces rectal radiation dose in patients receiving prostate SBRT and is associated with a decreased rate of acute GI toxicity.

## Introduction

Stereotactic body radiation therapy (SBRT) is a recommended treatment for prostate cancer and is increasingly utilized (1, 2). This technique, which utilizes ultra-hypofractionated radiation regimens (≥ 5 Gy per fraction), is now standard of care and has been suggested to be non-inferior to standard fractionation radiation for biochemical and local control (3). Additionally, ultra-hypofractionated treatment courses with SBRT, which require only 5–7 visits, are significantly more convenient for patients. However, gastrointestinal (GI) toxicity remains an issue for prostate SBRT. For example, the PACE-B trial reported 53% Grade 1, 10% Grade 2, and < 1% Grade 3 Radiation Therapy Oncology Group (RTOG) GI toxicities (4). The rectum is adjacent to and often abuts the prostate and thus may receive significant incidental radiation leading to GI toxicity. Acute radiation-related rectal toxicity can occur due to inflammation, fibrosis, microvascular damage, and edema within the bowel wall and mucosa (5, 6). Late sequelae may include bleeding, urgency, and incontinence, which can be predicted by radiation volumetric dose parameters (7–10).

**Figure 1:**
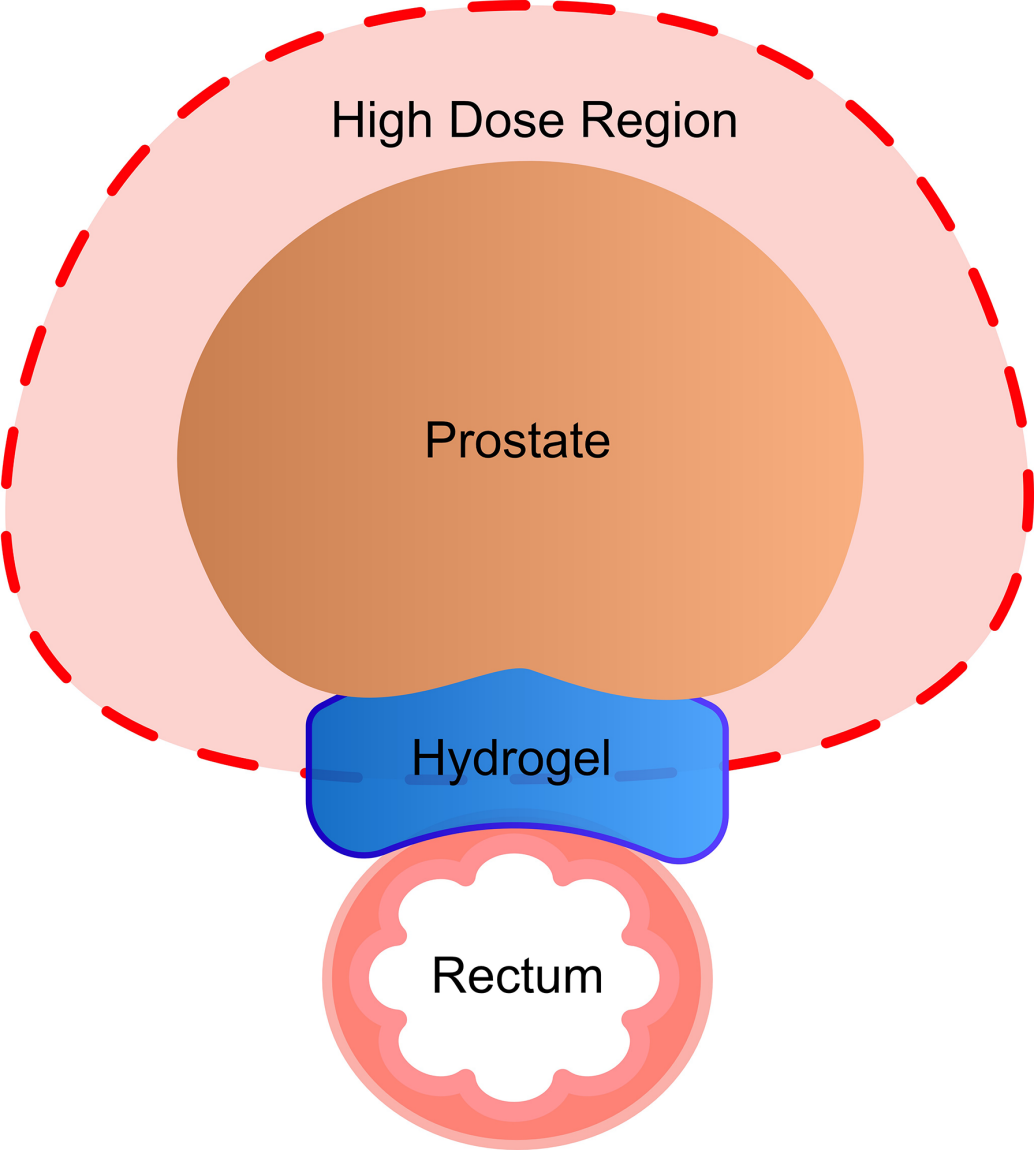
The hydrogel pushes the rectum out of high-dose radiation field.

To limit radiation dose to the rectum, various methods have been employed to create space between the prostate and rectum, including collagen or hyaluronic acid injection, and biodegradable rectal spacer balloons (11–13). Another such method is the injection of the hydrogel into Denonvilliers’ fascia between the rectum and prostate. This hydrogel is biologically inert and composed of two liquids that mix post-injection to polymerize and solidify within the patient (**Figure 1**). Hydrogel has been shown to reduce rectal dose in patients receiving standard fractionation radiation therapy (14). However, data on the safety, efficacy, and clinical outcomes of hydrogel in patients receiving SBRT are limited.

The purpose of this study is to compare rectal dose and associated GI toxicity with or without hydrogel in patients with prostate cancer undergoing SBRT to the prostate (Boston Scientific, Marlborough, MA, USA).

## Methods

This Institutional Review Board-approved retrospective study included patients who received SBRT for treatment of localized prostate cancer at a single academic institution between February 2017 and January 2020. All patients were aged 18 years or older and did not receive prior pelvic radiation, transurethral resection of the prostate, or any other focal treatment.

Hydrogel was offered to all patients without posterior extracapsular extension (ECE) on MRI. For patients receiving hydrogel, Denonvilliers’ space was approached transperineally with a 17-gauge needle and was gently hydrodissected with 10 cm^3^ of 0.9% normal saline under transrectal ultrasound guidance. Upon confirmation of Denonvilliers’ space expansion and separation of Denonvilliers’ fascia from the rectal wall, 10 cm^3^ of hydrogel was administered into this space. All patients underwent pretreatment multiparametric MRI at diagnosis and CT simulation for SBRT treatment planning. Patients who received hydrogel also subsequently underwent MRI within 1 week of CT simulation. All patients received linear accelerator-based radiation treatment with 40 Gy in 5 fractions to the clinical target volume (CTV), which was defined as the prostate and proximal seminal vesicles. The CTV was expanded by 5 mm in all directions, except 3–4 mm posteriorly to form the planning target volume (PTV). The treatment dose was prescribed such that 95% of the PTV received the prescription dose, and the institutional dose constraints were rectum V20Gy ≤ 50%, V32Gy ≤ 20%, V36Gy ≤ 10%, and V40Gy ≤ 5%; bladder V20Gy ≤ 40% and V40Gy ≤ 10%; and small bowel V20Gy < 30 cc and D0.035cc ≤ 35 Gy.

Baseline characteristics including age, body mass index (BMI), prostate volume, rectal diameter, T-stage, Gleason Grade Group, pretreatment prostate-specific antigen (PSA), and androgen deprivation therapy (ADT) use were collected. Risk categories were defined according to the National Comprehensive Cancer Network (NCCN). Prostate volume was assessed on pretreatment MRI, and rectal diameter was measured as the largest diameter at the mid-gland level of the prostate on the CT simulation scan.

The age, BMI, prostate volume, rectal diameter, and pretreatment PSA between the hydrogel and non-hydrogel patients were compared by Student’s or Welch’s t-test. The Gleason scores and risk groups were compared using the Kruskal–Wallis test, while T-stage and ADT use were compared using Fisher’s exact test.

Rectal dose–volume histogram (DVH) parameters corresponding to institutional dose constraints (rectum V40Gy, V36Gy, V32Gy, and V20Gy) and V38Gy(cc), which has previously been shown to predict high-grade late hematochezia, were collected (15). Differences between the hydrogel and non-hydrogel patients in rectal dose parameters were compared using t-test for two-sample mean when variances between groups were equal and Welch’s test when unequal, and the proportion of patients with V38Gy < 2 cc was compared using Fisher’s exact test. The highest reported Common Terminology Criteria for Adverse Events (CTCAE) for acute (≤ 90 days) and late (> 90 days) GI toxicity scores reported during follow-up were collected and compared using Fisher’s exact test. Posttreatment PSAs were collected to evaluate the incidence of biochemical recurrence per Phoenix definition (PSA nadir +2 ng/ml).

## Results

A total of 92 localized prostate cancer patients were identified who underwent SBRT, of whom 51 patients received hydrogel. Baseline characteristics are shown in **Table 1**, and no significant differences were observed (**Table 1**). The median overall follow-up was 14.8 months (range 3.8–41.5 months; hydrogel median 14.8 months, non-hydrogel median 16.2 months), and the median age was 72 years (range 46–85). Included in the study were 20 high-risk, 65 intermediate-risk, and 7 low-risk patients defined by NCCN criteria. A trend towards NCCN high-risk group disease in non-hydrogel patients and towards unfavorable intermediate-risk group disease in hydrogel patients was observed but was not statistically significant. A total of 3 patients (2 hydrogel patients) had T3a disease. None of these patients had posterior ECE on imaging. A total of four patients (1 hydrogel patient) had T3b disease. Androgen deprivation therapy was given to 39% and 35% of the hydrogel and non-hydrogel patients, respectively. The median time from hydrogel placement to SBRT was 10 days (range 4–25 days). At a median follow-up of 14.8 months in the hydrogel group, there were no biochemical recurrences.

**Table 1:** Baseline patient clinical characteristics.

	No hydrogel (n = 41)	Hydrogel (n = 51)	p-Value
Characteristics	Number of patients (%)		
Age			0.77
≤60	6 (15%)	6 (12%)	
61–70	12 (29%)	15 (29%)	
≥70	23 (56%)	30 (59%)	
	Median = 71 (range 46–85)	Median = 72 (range 52–85)	
BMI (kg/m^2^)	Mean = 26.9, median = 26.4 (range 20.2–45.0)	Mean = 26.7, median = 26.4 (range 16.1–35.6)	0.77
Stage			0.70
T1–T2	37 (90%)	48 (94%)	
T3 and above	4 (10%)	3 (6%)	
Grade group			0.81
1	3 (7%)	5 (10%)	
2	17 (41%)	16 (32%)	
3	11 (27%)	22 (44%)	
4	5 (12%)	5 (10%)	
5	5 (12%)	2 (4%)	
Pretreatment PSA (ng/ml)			0.82
< 10	27 (66%)	40 (78%)	
10–20	11 (27%)	10 (20%)	
> 20	3 (7%)	1 (2%)	
	Mean = 11.4, median = 7.64, (range 2.5–77)	Mean = 12.6, median = 7.1 (range 0.9–254.4)	
ADT			0.83
Yes	16 (39%)	18 (35%)	
No	25 (61%)	31 (65%)	
	Median = 6 months, (range 3–24 months)	Median = 6 months, (range 1.5–24 months)	
NCCN risk category			0.25
Low	2 (5%)	5 (10%)	
Favorable intermediate	13 (32%)	10 (20%)	
Unfavorable intermediate	14 (34%)	28 (55%)	
High	12 (29%)	8 (16%)	
Prostate volume (cc)	Mean = 56.6, median = 52.2 (range 27.3–112.3)	Mean = 49.1, median = 45.7 (range 16.5–86.8)	0.07
Rectal diameter (cm)	Mean = 3.7, median = 3.5 (range 2.4–5.3)	Mean = 3.6, median = 3.5 (range 2.3–5.6)	0.54

BMI, body mass index; PSA, prostate-specific antigen; ADT, androgen deprivation therapy; NCCN, National Comprehensive Cancer Network.

**Figure 2:**
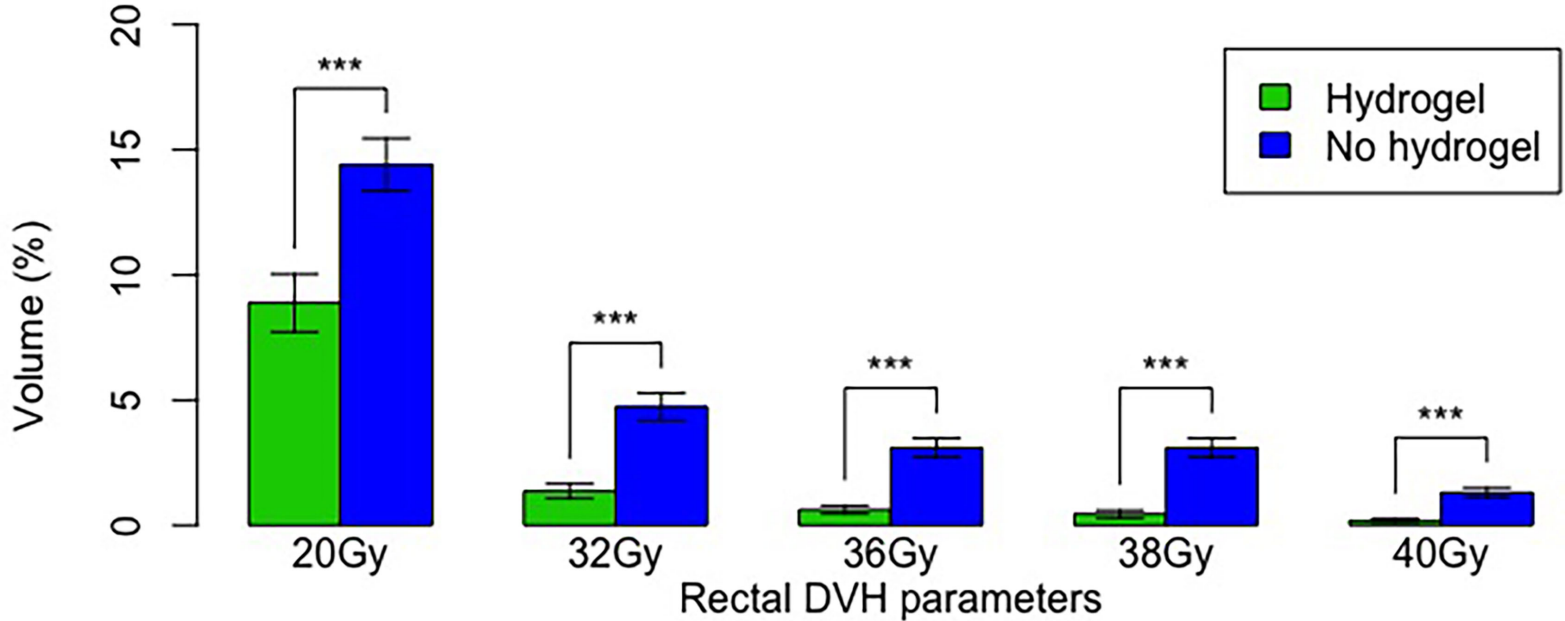
Radiation dose parameters in the hydrogel and non-hydrogel cohorts. Error bars show standard error. “***” denotes p < 0.001.

Rectal dose was significantly lower for all evaluated radiation dose parameters in the hydrogel group (**Figure 2**). The greatest relative differences were seen in the high dose parameters; i.e., V40Gy was 7-fold less in the hydrogel group (0.18% vs. 1.30%). Additionally, rectal V38(cc) was significantly less in the hydrogel group (mean 0.44 vs. mean 1.41 cc, p = 0.0002), and the proportion of patients with V38(cc) < 2 cc was greater in the hydrogel group (92% vs. 72%, p = 0.01).

The rates of acute Grade 1, 2, and 3 GI toxicities are shown in **Table 2**. Overall, the non-hydrogel group had greater acute GI toxicity (p = 0.006), including rectal urgency (4), constipation (2), and diarrhea (4). While most of the toxicities were Grade 1, one case of constipation was Grade 2, and one case of diarrhea was Grade 3, which later resolved. Of note, 6 minor acute Grade 1 adverse events resulting from the procedure were reported in the current cohort, and all resolved, including constipation (2), loose stools (1), and minimal or unspecified GI symptoms (3). The highest reported late GI toxicity was Grade 1 (diarrhea in all cases): 2 patients (4%) in the hydrogel group and 4 patients (10%) in the non-hydrogel group. This difference in late GI toxicity was not statistically significant (p = 0.219).

**Table 2:** Acute GI toxicity rates.

	Non-hydrogel % (n)	Hydrogel % (n)
Grade 1	24% (10)	12% (6)
Grade 2	2% (1)	4% (2)
Grade 3	2% (1)	0% (0)

GI, gastrointestinal.

## Discussion

Hydrogel significantly reduced the relevant radiation volumetric dose parameters by creating a physical separation between the prostate and rectum and thereby displacing the rectum from the high dose radiation field. The procedure was safe and well tolerated with no short- or long-term procedural-related sequelae. Furthermore, hydrogel was associated with a significant reduction in acute GI toxicity. We did not observe a similar association for late GI toxicity; however, more events may occur with longer follow-up. The acute diarrhea reported in the hydrogel group may also be due to hydrogel, and not radiation, given that hydrogel may irritate the rectum and that patients are often prescribed a stool softener to prevent constipation.

Importantly, there were no differences in biochemical recurrence, indicating oncologic outcomes were not compromised. However, the median overall follow-up time was only 14.8 months. Furthermore, there were only three patients with ECE (none were posterior) and four patients with seminal vesicle invasion. Therefore, caution should be exercised for T3–T4 patients with posterior ECE or invasion of the rectum out of theoretical concern that gross disease may be displaced out of the intended treatment field. Therefore, pretreatment MRI is recommended to assess disease extent posteriorly.

Though data on the effects of hydrogel in patients receiving SBRT are limited, the dose reduction observed in this study is consistent with a previous study of hydrogel with dose-escalated standard fractionation radiation (14), which similarly showed the greatest relative reductions in the high dose volumetric parameters; i.e., V82Gy was also 7-fold less in the hydrogel group (0.2% vs. 1.3%). Though no difference in acute GI toxicity was reported, late Grade 1 toxicity was less frequent in the hydrogel group (16.6% vs. 41.8%). Another study of hydrogel with ultra-hypofractionation without a comparative non-hydrogel group showed similar rates of acute GI toxicity (16% Grade 1 and 4% Grade 2) and no difference in late rectal toxicity (16). Studies of MRI-guided, daily adaptive SBRT similarly show reduced rectal dose and reduced intra-fraction motion and importantly collected patient-reported outcomes that did not show decreased quality of life in patients receiving hydrogel spacers (17, 18). The acute benefit of hydrogel may be more pronounced for ultra-hypofractionation than for standard fractionation, especially given the concern for the worse acute quality of life for ultra-hypofractionation seen in the HYPO-RT-PC trial (19). Acute Bowel Quality of Life was worse at <3 months but the same at 3 months. Furthermore, the HYPO-RT-PC SBRT arm reported 9.4% acute Grade 2+ and 2.2% late 2-year Grade 2+ GI toxicity rates. The higher rate of Grade 2+ toxicity on HYPO-RT-PC compared to the current study may be due to the use of older radiation techniques. Additionally, the main rectal dose constraint used in the HYPO-RT-PC trial was V90% ≤ 15%, while our corresponding institutional constraint was V36Gy ≤ 10%. A phase II trial of SBRT at our institution demonstrated that acute and late Grade 2 GI toxicities were 3.3% and 3.9%, respectively (20).

It is imperative that the risks and benefits of hydrogel be considered prior to its administration. Hall et al. queried the Food and Drug Administration (FDA) Manufacturer and User Facility Design Database (MAUDE) and noted 85 adverse events related to hydrogel placement, of which 69% were scored as grade ≥ 3 toxicity including descriptions of colostomy, anaphylactic events, rectal injection pulmonary emboli, and death (21). There is no doubt that these events are severe, but in relation to the total number of hydrogel cases performed (109,165 estimated), these events are rare (0.07%). Of note, adequate training and experience are critical to ensure the safety of hydrogel administration, and physicians must be credentialed to perform this procedure.

The current study has several limitations, one of which is that this is a retrospective analysis of non-randomized patients with a contemporary control used instead. Furthermore, physician preference and insurance reimbursement may have driven the decision patient decision to pursue hydrogel, and these confounders may be correlated with toxicity outcomes. Hydrogel patients were only simulated once without a separate plan to compare DVH parameters without hydrogel within the same patient. Furthermore, physician-reported toxicity may underestimate the true incidence of GI toxicities, and late GI Grade 2+ GI toxicity occurs with a mean time of 1.5 years posttreatment, which exceeds the median follow-up of the current study (22, 23).

## Conclusion

In prostate cancer patients treated with SBRT, hydrogel is well tolerated, reduced key rectal dose parameters, and is associated with lower rates of acute GI toxicity.

## Data Availability Statement

The data analyzed in this study is subject to the following licenses/restrictions: due to the nature of this research and protected health information, participants of this study did not agree for their data to be shared publicly. Requests to access these datasets should be directed to AC, ajchang@mednet.ucla.edu.

## Author Contributions

All authors contributed equally to the writing and preparation of this manuscript.

## Conflict of Interest

AC received consulting fees and lecture payments from Boston Scientific.

The remaining authors declare that the research was conducted in the absence of any commercial or financial relationships that could be construed as a potential conflict of interest.

## Publisher’s Note

All claims expressed in this article are solely those of the authors and do not necessarily represent those of their affiliated organizations, or those of the publisher, the editors and the reviewers. Any product that may be evaluated in this article, or claim that may be made by its manufacturer, is not guaranteed or endorsed by the publisher.
